# A Cross-Sectional Study on Bedside Abdominal Ultrasound Findings as a Diagnostic and Prognostic Tool in Dengue Fever in Manipal Hospital, Bengaluru, India

**DOI:** 10.7759/cureus.64734

**Published:** 2024-07-17

**Authors:** Abhishek Jain, Mabel Vasnaik, Amol Singam, V. N. K. Srinivas Mudiganti

**Affiliations:** 1 Critical Care Medicine, Jawaharlal Nehru Medical College, Datta Meghe Institute of Higher Education and Research, Wardha, IND; 2 Emergency Medicine, Manipal Hospital, Bengaluru, IND

**Keywords:** bengaluru, manipal hospital, prognostic tool, diagnostic tool, bedside ultrasound, dengue fever

## Abstract

Background

Dengue fever poses a significant health burden globally, particularly in tropical and subtropical regions. Early diagnosis and effective management are crucial in reducing morbidity and mortality associated with the disease. Bedside abdominal ultrasound has emerged as a promising tool for assessing dengue patients, providing real-time imaging of abdominal organs, and aiding clinical decision-making.

Materials and methods

This cross-sectional study was conducted in 55 adult emergency departments of Manipal Hospital, Bengaluru, from March 2017 to March 2018. Adult patients presenting with signs and symptoms suggestive of dengue fever were included. Clinical data, laboratory investigations, and bedside abdominal ultrasound findings were systematically recorded and analyzed using appropriate statistical methods.

Results

Descriptive statistics revealed characteristic clinical measurements and symptom ratings observed in dengue fever patients. Frequency distributions highlighted common symptoms encountered, while statistical analyses demonstrated significant associations between ultrasonic parameters, disease severity, and outcomes. The study found notable correlations between ultrasonic findings and dengue severity levels, emphasizing the potential of bedside ultrasound as a diagnostic and prognostic tool.

Conclusion

Bedside abdominal ultrasound shows promise as a valuable adjunctive tool in assessing dengue fever patients. The significant associations between ultrasonic parameters and disease severity suggest its utility in risk stratification and guiding clinical management decisions. Incorporating bedside ultrasound into routine practice may improve patient care and outcomes in dengue fever management. Further research is warranted to validate these findings and explore additional bedside ultrasound applications in dengue fever diagnosis and prognosis.

## Introduction

Dengue fever, caused by the dengue virus and transmitted primarily by Aedes mosquitoes, is a significant global health concern, particularly in tropical and subtropical regions. The disease is endemic in over 100 countries, with an estimated 390 million infections occurring annually worldwide [1-3]. Dengue fever presents a spectrum of clinical manifestations, ranging from mild febrile illness to severe forms, including dengue hemorrhagic fever (DHF) and dengue shock syndrome (DSS), which can lead to life-threatening complications such as plasma leakage, hemorrhage, and organ failure [4].

Early diagnosis and prompt clinical management are crucial in reducing morbidity and mortality associated with dengue fever [5]. However, diagnosing dengue fever based solely on clinical symptoms can be challenging due to its nonspecific presentation, which overlaps with other febrile illnesses such as malaria and chikungunya. Laboratory confirmation through serological tests or molecular diagnostics is essential for accurate diagnosis [6].

In recent years, bedside ultrasound has emerged as a valuable adjunctive tool in the clinical assessment of dengue fever patients [7]. Bedside ultrasound allows real-time imaging of abdominal organs, enabling clinicians to detect early signs of plasma leakage and assess disease severity. Studies have shown that ultrasound findings such as gall bladder wall thickening, ascites, hepatomegaly, splenomegaly, and pleural effusion are associated with dengue severity and can aid in risk stratification and prognostication [8,9].

Manipal Hospital, Bengaluru, is a tertiary care center that serves a large population in southern India. Given the high burden of dengue fever in this region, there is a need to evaluate the utility of bedside abdominal ultrasound as a diagnostic and prognostic tool in dengue fever management. This cross-sectional study assesses the correlation between bedside abdominal ultrasound findings and disease severity in adult patients with dengue fever symptoms at Manipal Hospital, Bengaluru.

## Materials and methods

Study setting and design

The study was conducted in 55 adult emergency departments of Manipal Hospital in Bengaluru. It employed a cross-sectional research design, covering the period from March 2017 to March 2018.

Study population

The study included adult patients who presented with signs and symptoms indicative of dengue fever. Inclusion criteria encompassed individuals over 18 years old with symptoms such as fever lasting three to four days, headache, retro-orbital pain, nausea, vomiting, joint and muscle aches, leucopenia, rashes, abdominal pain, loose stools, and thrombocytopenia. Exclusion criteria comprised patients diagnosed with malaria, acalculous cholecystitis, liver disease, portal hypertension, abdominal cancer, heart failure, or those unfit for bedside ultrasound scans.

Data collection

Data collection involved the systematic recording of patient symptoms, vital signs, and clinical examination findings using the data collection form (given in Appendices). Bedside ultrasonography was performed on eligible patients using the LOGIQ P9 ultrasound machine (GE Healthcare, Chicago, USA) equipped with a curvilinear probe with a frequency of 4 MHz. Ultrasound examinations evaluated gall bladder thickness, splenomegaly, ascites, hepatomegaly, pericardial, and pleural effusion. Laboratory investigations included hematocrit, leucopenia, thrombocytopenia, liver enzyme levels, and dengue serology (dengue NS1 antigen and IgM/IgG antibodies).

Statistical analysis

The data obtained were analyzed using appropriate statistical methods. Descriptive statistics such as means, standard deviations, frequencies, and percentages were used to summarize patient characteristics and ultrasound findings. Inferential statistics, such as Chi-square tests or logistic regression, were employed to explore associations between ultrasound findings, laboratory parameters, and disease outcomes.

Ethical consideration

Ethical considerations were paramount throughout the study. Informed consent was obtained from all participants before inclusion in the study. Patient confidentiality was strictly maintained, and data were anonymized to ensure privacy. Manipal Hospital, Bengaluru's institutional ethics committee reviewed and approved the study protocol before commencement (reference number: 152-27135-161-210803).

## Results

Table 1 provides an overview of the clinical measurements and symptom ratings observed in patients with dengue fever.

**Table 1:** Descriptive statistics over symptom scale and vitals parameters HR: heart rate; RR: respiratory rate; SBP: systolic blood pressure; DBP: diastolic blood pressure

Parameter	Mean	Median	Std. Deviation
Temperature (°F)	100.8600	100.0000	0.80866
Headache Pain	4.6909	5.0000	1.93288
Body Ache Scale	4.7636	5.0000	2.14272
Abdominal Pain	4.5636	5.00000	2.00706
HR (Per Min)	116.0182	116.0000	9.53840
RR (Per Min)	17.7091	18.0000	3.81844
SBP	99.2909	99.0000	17.88199
DBP	60.7091	60.0000	15.04890

Table 2 lists the frequency and percentage of specific symptoms encountered in dengue patients, such as rash, vomiting, pericardial effusion, and lethargy.

**Table 2:** Frequency distribution of symptoms

Symptoms	Frequency	Percentages
Rash	10	18.2%
Vomiting	38	69.1%
Pericardial Effusion	4	7.3%
Lethargy	38	69.1%

Table 3 presents an analysis of variance (ANOVA) of ultrasonic parameters such as gall bladder wall thickness, hepatomegaly, and splenomegaly across different severity levels (mild, moderate, severe) of dengue.

**Table 3:** Comparative assessment of severity of dengue over ultrasonic parameters **: significant; GBWT: gallbladder wall thickness

Condition	Group	N	Mean	Std. Deviation	Std. Error	95% CI Lower Bound	95% CI Upper Bound	Minimum	Maximum	F-value	P-value
GBWT above 3 mm	Mild	21	3.35	0.21	0.047	3.25	3.44	3.00	3.80	299.65	<0.001**
	Moderate	23	3.85	0.13	0.02	3.79	3.90	3.60	4.20		
	Severe	11	5.08	0.23	0.07	4.92	5.24	4.70	5.40		
Hepatomegaly	Mild	21	12.28	1.10	0.24	11.78	12.79	10.00	15.00	4.15	<0.001**
	Moderate	23	13.14	1.93	0.40	12.30	13.97	10.00	16.00		
	Severe	11	14.03	1.91	0.57	12.74	15.31	10.90	15.90		
Splenomegaly	Mild	21	10.55	0.32	0.07	10.40	10.69	10.00	11.20	11.99	<0.001**
	Moderate	23	10.99	0.87	0.18	10.61	11.37	10.20	12.90		
	Severe	11	12.00	1.18	0.35	11.20	12.80	10.23	13.30		

Table 4 evaluates the severity of dengue through different ultrasonic findings, such as pericardial effusion, ascites, and pleural effusion, categorized by severity levels using a Chi-square test.

**Table 4:** Severity of dengue over ultrasonic parameters at different categories using Chi-square test **: significant

Parameter	Category	Mild	Moderate	Severe	Chi Sq	P-value
Pericardial Effusion	No	21 (100%)	23 (100%)	7 (63.6%)	17.255	<0.0001**
Ascites	No	17 (81%)	9 (39.1%)	2 (18.2%)		
	Mild	4 (19%)	0 (0%)	0 (0%)		
	Moderate	0 (0%)	14 (60.9%)	2 (18.2%)	55.68	<0.001**
	Severe	0 (0%)	0 (0%)	7 (63.6%)		
Pleural Effusion	No	21 (100%)	13 (56.5%)	1 (9.1%)		
	Unilateral	0 (0%)	8 (34.8%)	4 (36.4%)	32.80	<0.001**
	Bilateral	0 (0%)	2 (8.7%)	6 (54.5%)		

Table 5 details the statistical analysis correlating dengue severity with ultrasonic scores.

**Table 5:** Dengue severity and ultrasonic parameters **: significant

Severity Level	Mean	Std. Deviation	Std. Error	95% Confidence Interval	Minimum	Maximum	F-value	P-value
Mild	26.37	1.35	0.29	25.76 – 26.99	23.3	30.1	102.412	<0.01**
Moderate	29.73	1.89	0.39	28.91 – 30.55	25.1	34.4		
Severe	35.20	1.66	0.50	34.09 – 36.32	32.44	38.4		

## Discussion

The findings of this cross-sectional study highlight the potential of bedside abdominal ultrasound as a valuable diagnostic and prognostic tool in managing dengue fever. The significant correlations observed between ultrasonic parameters and dengue severity levels are in line with previous research, affirming the growing recognition of bedside ultrasound's utility in evaluating dengue patients [10-12]. Bedside ultrasound plays a crucial role in detecting fluid accumulation and organ involvement, which are key indicators of disease progression. These findings emphasize the importance of integrating ultrasound into routine clinical practice for early identification of severe cases and timely intervention. Early recognition of complications such as plasma leakage and hemorrhage is critical in preventing adverse outcomes and reducing mortality rates associated with severe dengue [13,14].

Despite the promising results, it is essential to acknowledge the limitations of our study. The cross-sectional design restricts our ability to establish causality or assess long-term outcomes. Additionally, the study's single-center nature may limit the generalizability of the findings. Future research should prioritize multicenter studies with larger sample sizes to validate our results and further explore the diagnostic and prognostic utility of bedside ultrasound in diverse patient populations [15,16].

Despite these limitations, the findings of this study have significant clinical implications. Bedside abdominal ultrasound offers a non-invasive, rapid, and cost-effective means of assessing dengue patients, particularly in resource-limited settings where advanced imaging modalities may not be readily available [17]. Integrating ultrasound into dengue management protocols can improve patient outcomes and reduce healthcare costs associated with unnecessary interventions or delayed diagnoses [18,19]. This study underscores the importance of bedside abdominal ultrasound in dengue fever management and highlights the need for further research to fully realize its potential in enhancing patient care and outcomes.

## Conclusions

In conclusion, this study underscores the potential of bedside abdominal ultrasound as a valuable adjunctive tool in assessing and managing dengue fever. The significant correlations between ultrasonic parameters and disease severity levels highlight its utility in risk stratification and guiding clinical decision-making by providing real-time imaging of abdominal organs, bedside ultrasound aids in the early detection of complications such as ascites, pleural effusion, and pericardial effusion, facilitating timely intervention and improving patient outcomes. Further research is warranted to validate these findings across diverse patient populations and explore additional bedside ultrasound applications in the broader spectrum of infectious diseases.

## Appendices

Appendix I: Data collection form

Patient Information

Patient ID: ____________________

Age: ____________________

Gender: ____________________

Date of Admission: ____________________

Date of Discharge: ____________________

Symptoms and Clinical History

Fever (days): ____________________

Headache: Yes/No

Retro-orbital Pain: Yes/No

Nausea/Vomiting: Yes/No

Joint Pain: Yes/No

Muscle Ache: Yes/No

Rash: Yes/No

Abdominal Pain: Yes/No

Loose Stools: Yes/No

Lethargy: Yes/No

Vital Signs

Temperature (°F): ____________________

Heart Rate (beats per minute): ____________________

Respiratory Rate (breaths per minute): ____________________

Systolic Blood Pressure (mmHg): ____________________

Diastolic Blood Pressure (mmHg): ____________________

Laboratory Investigations

Hematocrit (%): ____________________

Leucopenia: Yes/No

Platelet Count (per µL): ____________________

Liver Enzyme Levels (ALT/AST): ____________________

Dengue Serology (NS1/IgM/IgG): Positive/Negative

Ultrasound Findings

Gallbladder Wall Thickness (mm): ____________________

Ascites: None/Mild/Moderate/Severe

Hepatomegaly (liver span in cm): ____________________

Splenomegaly (spleen span in cm): ____________________

Pericardial Effusion: Yes/No

Pleural Effusion: None/Unilateral/Bilateral

Additional Clinical Findings

Signs of Plasma Leakage: Yes/No

Hemorrhagic Manifestations: Yes/No

Other Complications: ____________________

Summary of Clinical Course

Initial Severity (Mild/Moderate/Severe): ____________________

Final Outcome (Recovered/Complications/Deceased): ____________________

Length of Hospital Stay (days): ____________________

Investigator Details

Name of Investigator: ____________________

Signature: ____________________

Date: ____________________
